# Collection and processing of whole blood for transformation of peripheral blood mononuclear cells and extraction of DNA: the Type 1 Diabetes Genetics Consortium

**DOI:** 10.1177/1740774510373493

**Published:** 2010-08

**Authors:** Silke Rosinger, Sarah Nutland, Eric Mickelson, Michael D Varney, Bernard O Boehm, Gary J Olsem, John A Hansen, Ian Nicholson, Joan E Hilner, Letitia H Perdue, June J Pierce, Beena Akolkar, Concepcion Nierras, Michael W Steffes, the T1DGC

**Affiliations:** ^a^Division of Endocrinology and Diabetes, Department of Internal Medicine, Ulm University, Ulm, Germany, ^b^JDRF/Wellcome Trust Diabetes and Inflammation Laboratory, Cambridge Institute for Medical Research, University of Cambridge, Cambridge, UK, ^c^Human Immunogenetics Program, Fred Hutchinson Cancer Research Center, Seattle, WA, USA, ^d^Victorian Transplantation and Immunogenetics Service (VTIS), Australian Red Cross Blood Services, Melbourne, Victoria, Australia, ^e^Department of Biostatistics, School of Public Health, University of Alabama at Birmingham, Birmingham, AL, USA, ^f^Division of Public Health Sciences, Wake Forest University Health Sciences, Winston-Salem, NC, USA, ^g^Division of Diabetes, Endocrinology and Metabolic Diseases, National Institute of Diabetes and Digestive and Kidney Diseases, National Institutes of Health, Bethesda, MD, USA, ^h^Juvenile Diabetes Research Foundation International, New York, NY, USA, ^i^Department of Laboratory Medicine and Pathology, University of Minnesota Medical School, Minneapolis, MN, USA

## Abstract

***Background and Purpose*** To yield large amounts of DNA for many genotype analyses and to provide a renewable source of DNA, the Type 1 Diabetes Genetics Consortium (T1DGC) harvested DNA and peripheral blood mononuclear cells (PBMCs) from individuals with type 1 diabetes and their family members in several regions of the world.

***Methods*** DNA repositories were established in Asia-Pacific, Europe, North America, and the United Kingdom. To address region-specific needs, different methods and sample processing techniques were used among the laboratories to extract and to quantify DNA and to establish Epstein-Barr virus transformed cell lines.

***Results*** More than 98% of the samples of PBMCs were successfully transformed. Approximately 20–25 µg of DNA were extracted per mL of whole blood. Extraction of DNA from the cell pack ranged from 92 to 165 µg per cell pack. In addition, the extracted DNA from whole blood or transformed cells was successfully utilized in each regional human leukocyte antigen genotyping laboratory and by several additional laboratories performing consortium-wide genotyping projects.

***Limitations*** Although the isolation of PBMCs was consistent among sites, the measurement of DNA was difficult to harmonize.

***Conclusions*** DNA repositories can be established in different regions of the world and produce similar amounts of high-quality DNA for a variety of high-throughput genotyping techniques. Furthermore, even with the distances and time necessary for transportation, highly efficient transformation of PBMCs is possible. For future studies/trials involving several laboratories in different locations, the T1DGC experience includes examples of protocols that may be applicable. In summary, T1DGC has developed protocols that would be of interest to any scientific organization attempting to overcome the logistical problems associated with studies/trials spanning multiple research facilities, located in different regions of the world.

## Introduction

Type 1 diabetes is a multi-factorial autoimmune disease in which the insulin producing β-cells are selectively destroyed [1]. The etiology of type 1 diabetes is only partially characterized; however, it is generally accepted that a certain genetic predisposition as well as environmental impacts [2] increase the risk to develop the disease. Furthermore, it is recognized that type 1 diabetes is strongly clustered in families [3]. The Type 1 Diabetes Genetics Consortium (T1DGC) brought together several groups of investigators worldwide who shared the common goal of identifying genes related to the etiologies of type 1 diabetes. With the advent of high-throughput instrumentation to process the DNA for its genetic information, more research groups propose utilizing the DNA from clinical studies and trials. A study involving large numbers of subjects and laboratories needs to harmonize the methods used to process and distribute the DNA to genotyping facilities.

From its inception, the T1DGC developed a worldwide strategy with goals to collect whole blood and to develop transformed cell lines from peripheral blood mononuclear cells (PBMCs) for current or later extraction of the DNA. The T1DGC repositories extracted DNA and provided the samples to laboratories for human leukocyte antigen (HLA) typing and genotyping. Furthermore, the need for a rapid processing of the samples mandated establishment of repositories in several different locations: Asia-Pacific, Europe, North America, and the United Kingdom.

Reliable measurement of DNA concentration is important for many applications in molecular biology. Common techniques that use DNA, such as polymerase chain reaction (PCR), sequencing, complementary DNA (cDNA) synthesis, and cloning, all benefit from an accurately defined template concentration. The most common reasons for failure are low quantity and poor quality of the DNA. The processes and protocols underlying efforts of the T1DGC to provide adequate amounts of high-quality DNA are described in this article.

## Methods

### Repositories

To address the needs of the clinical centers throughout the world, repositories were established in Asia-Pacific (Australian Red Cross Blood Service, Melbourne, Australia), Europe (Ulm University, Ulm, Germany), North America (Fred Hutchinson Cancer Research Center, Seattle, WA, USA), and the United Kingdom (Cambridge University, Cambridge, UK).

### Participants

Worldwide recruitment of the population is described by Rich et al. [4] and Hilner et al. [5]. The fundamental need to recruit participants from several geographic areas informed the decisions to establish network-specific repositories, thereby allowing efficient shipping of whole blood samples. Within each region, procurement and processing of samples from probands and their relatives were identical. To minimize any differences in the collection and processing of samples, all blood collection tubes and fetal bovine serum (FBS) were obtained from centralized sources (Sarstedt, Inc and Invitrogen, Inc., respectively) as described by Hilner et al. [5]. A central source of the United States Department of Agriculture (USDA) approved FBS also was required to enable subsequent transfer of the samples to a central repository in the United States.

### Isolation and transformation of PBMCs

PBMCs were isolated from whole blood by standard ficoll–hypaque density gradient centrifugation. Briefly, approximately 10 mL of heparinized, plasma-reduced blood was diluted with Hank’s buffered salt solution (HBSS; 1:2 dilution). Then, 15 mL of ficoll was covered with a layer of diluted blood (30 mL). After 30 min of centrifugation (2000 rpm, room temperature (RT), without break), the PBMCs could be easily collected. After two washing steps and cell counting, the PBMCs were prepared for transformation with Epstein-Barr virus (EBV) added directly after isolation of the PBMCs (Asia-Pacific and European DNA Repositories); or the isolated PBMCs were frozen and stored in a liquid nitrogen freezer for future batch transformation (North American and United Kingdom DNA Repositories). PBMCs were frozen in FBS containing 10% dimethylsulfoxide (DMSO). The protocols for transformation of cells were quite similar whether the PBMCs were transformed after isolation or after storage in liquid nitrogen (details are provided below).

For transformation of previously frozen PBMCs, the cells were thawed and washed in 10 mL of prewarmed HBSS to remove all traces of the cryoprotectant in the freezing medium. Following centrifugation at ∼300 × *g* for 5 min, the supernatant was discarded; the pellet was then resuspended in 1 mL of complete medium (RPMI 1640, 10–20% heat inactivated FBS, 1% penicillin–streptomycin, and 0.5% normocin or 0.1% gentamicin), and transferred to a 25 cm^2^ flask containing 1.0–2.0 mL of EBV supernatant and 1.0 µg of cyclosporine (CSA) per mL. Approximately 6–7 × 10^6^ cells were used for the transformation of both thawed and freshly isolated PBMCs.

Freshly isolated PBMCs were suspended in 14 mL of complete medium (RPMI 1640 with Glutamax, 10% heat inactivated FBS, 1% penicillin–streptomycin, and 0.5% normocin) in a 15 mL of Falcon tube, centrifuged at ∼350 × *g* for 10 min, and the supernatant was discarded. The cells were then re-suspended in 2.5 mL of EBV supernatant and 2.5 mL of complete medium, mixed carefully, incubated for at least 3 h (37°C; 6% CO_2_), and transferred to a 25 cm^2^ tissue culture flask. In the European, North American, and United Kingdom Repositories, CSA (at a final concentration of 1 µg/mL) was used to suppress growth of T-lymphocytes. The empty 15 mL Falcon tube was rinsed with 5 mL of CSA containing medium before transferring the CSA medium to the cells in the flask; and then the 10 mL flask was placed in the incubator. Alternatively, in Asia-Pacific, cells were re-suspended in 4.0 mL of complete medium supplemented with 5 mg/mL of phytohemagglutinin-M (PHA-M) instead of CSA and then transferred to a 25 cm^2^ tissue culture flask. EBV supernatant (1 mL) was added to the flask, mixed carefully, and then the flask was placed in a humidified incubator (37°C; 5% CO_2_).

The flasks were kept in a humidified incubator at 37°C and 5–6% CO_2_ throughout the culture period. They may be left undisturbed for the first 21 days, or may be subjected to additional procedures and/or observations during this time. In the latter case, on day 5, 0.3 mL of PHA solution (100 µg/mL) may be added to the flask to augment the suppression of T-lymphocytes. If the cultures were periodically examined during the first 3 weeks of incubation, they were first checked at day 5–7 by inverted phase microscopy for bright refractile clumps of cells (post-setup check). If there were a significant number of clumps present, 1–3 mL of complete medium (including 5 µL of CSA per mL) was added to the flask, depending on the number of clumps, and the flask was returned to the incubator. If there were very few clumps of cells visible, no medium was added, and the flask was returned to the incubator to allow further growth before repeating the post-setup check.

After 28–35 days of incubation, the cultures were checked for sufficient cell numbers and split into two portions, for freezing (one or more stock aliquots) or for DNA extraction. The remaining cells (1–20 mL, depending on final culture volume) were returned to a 25- or 75-cm^2^ flask for expansion to produce a sufficient number of cells for DNA extraction or freezing.

### Extraction of DNA

For whole blood samples, each laboratory first lysed red blood cells (RBCs) from a blood sample with reduced volume following removal of plasma. The remaining white blood cells were treated in a manner identical to that used on the cultured PBMCs to isolate DNA. Three laboratories (Asia-Pacific, Europe, and North America) used slight variations of the ‘salting out’ method [6] and one laboratory (United Kingdom) employed ‘chloroform’ extraction [7]. Briefly, as an example of the modified salting out method, the protocol of the European DNA Repository is described.

After freezing and fast thawing of the cell pack samples, the samples were diluted with cold phosphate buffered saline (PBS). After the addition of 3 volumes of ice-cold RBC lysis buffer (155 mM NH_4_Cl, 20 mM KHCO_3_, 0.1 mM Na_2_EDTA; pH 7.4), the RBCs are lysed after 15 min of incubation on ice followed by centrifugation (3000 rpm, 10 min, +4°C) and decanting. After an additional step of washing with RBC lysis buffer, the white cell pellet was then re-suspended in 5 mL of saline EDTA (SE) buffer (75 mM NaCl, 25 mM Na_2_EDTA; pH 8), 250 µL sodium dodecyl sulfate (SDS) (20%), and 5 µL of RNAse and incubated at 37°C for 15–30 min. After addition of 25 µL of proteinase K (20 µg/mL), the suspension was incubated overnight at 37°C and 130 rpm.

Next day, 5 mL of SE buffer is added, followed by 2–3 s of vortexing and at least 30 min of incubation at 55°C. After cooling to RT, 3 mL of saturated 6 M NaCl solution was added to the cell lysate, followed by immediate vortexing for exactly 25 s. To keep the SDS in the solution, the samples were then centrifuged at 22°C (3500 rpm, 10 min).

Following centrifugation, the supernatants were transferred to a 50-mL tube and one volume of 100% isopropanol was added. To precipitate the DNA, the samples were inverted gently 25 times. After an overnight incubation at −80°C, the samples were thawed at RT and centrifuged at 3500 rpm for 10 min at RT. The supernatants were decanted and the DNA was re-suspended in 5 mL of 70% ethanol and again centrifuged (3500 rpm, 10 min, RT). The supernatants were decanted and the DNA was air dried for 1.5–2 h at RT. The DNA was hydrated by adding Tris EDTA (TE) solution (TEKNOVA, # T022; volume is dependent of the strand size). To get better results of the DNA yield and concentration, the DNA solution was incubated for at least 1 h, at 55°C to ensure complete hydration.

As mentioned above, in the United Kingdom Network, DNA was extracted from blood with an in-house extraction protocol using chloroform. Initially, the red cells were lysed by washing twice with lysis buffer (320 mM sucrose, 1% Triton-X-100, 5 mM MgCl_2_, and 1 mM Tris-HCl; pH 7.4), followed by centrifugation (2500 rpm, 15 min) after each addition of the buffer. The remaining white cell pellet was digested overnight at 37°C with 4 mL of buffer (5.25 M GuHCl, 463 mM NH_4_Ac, 1.25% Na sarcosyl) and 50 µL of proteinase K (10 mg/mL). After cooling to RT, the extraction mix was transferred to 2 mL of chloroform and vortexed until a white emulsion formed. The tube was left to stand for 1 min before being centrifuged (2500 rpm, 3 min). The upper clear aqueous layer was removed and transferred to 10 mL of absolute ethanol. This tube was then incubated at −20°C for at least 1 h, preferably overnight. Following incubation, the tube was placed in a rotator for 5 min at 40 rpm to precipitate the DNA. The DNA was pelleted (3000 rpm for 15 min), and the ethanol was discarded. The pellet was washed with 2 mL of 70% ethanol followed by centrifugation (3000 rpm for 5 min). The ethanol was discarded and the pellet was left to air dry before being re-suspended in TE buffer (10 mM Tris, 0.1 mM EDTA).

### Measurement of DNA concentration

#### Absorbance

The most commonly used technique for measuring nucleic acid concentration, absorbance at 260 nm (A_260_), utilizes an average extinction coefficient for double-stranded (ds) DNA (1A_260_ = 50 µg/mL) to determine the nucleic acid concentration from the absorbance of the nucleic acid preparation. Absorbance at 280 nm (A_280_) permits estimation of protein concentration in the sample. The optimal density_260/280_ (OD_260/280_) ratio, reflecting the DNA and protein concentrations, should fall between 1.6 and 2.0 to confirm that the preparation is free from contamination with protein or RNA. For accurate results, the A_260_ should be in the range 0.05–0.10, which for a 1.0-mL assay, requires large amounts (2.5–5.0 µg) of dsDNA. For diluted nucleic acid samples, the solution being measured should be free of components that would add significantly to the absorbance at 260 nm. Limitations of the assay include: the large contribution of nucleotides; single-stranded nucleic acids and proteins to the signal; the interference caused by additional contaminants; and the inability to distinguish between DNA and RNA.

#### Fluorescence

Due to these limitations, alternate techniques have been sought to provide more sensitivity and less variation to the background absorbance. One such alternative for quantitation of DNA is fluorescence. PicoGreen® dsDNA quantitation reagent employs a ‘CytoFluor® Fluorescence Reader’, an eight-point standard curve (0.11–14 ng/µL), and three positive assay controls (5, 10, and 15 ng/µL). Samples are assayed in triplicate; standards and controls in duplicate with a coefficient of variation (CV) calculated for each set of readings. Samples and controls are excited at 480 nm with the fluorescence emission intensity measured at 520 nm. Fluorescence emission intensity plotted versus DNA concentration for the eight-point standard curve allows for the sample concentrations to be determined by extrapolating their fluorescence readings from the standard curve.

#### DNA concentration of stock solutions

Stock DNA samples were normalized, according to absorbance- or PicoGreen®-determined concentrations, to either 500 or 250 ng/µL or left at the determined concentration if measured to be lower than 250 ng/µL. To confirm the measured concentration by comparison with the control DNA sample, 2 µL of the normalized stock DNA sample was diluted to 100 ng/µL and run out on 0.75% agarose gel alongside a λ-Hind III ladder and a control DNA sample (Lambda-DNA; λ cl857 Sam 7; Roche) also at 100 ng/µL.

## Results

### Transformation of PBMCs

The different repositories utilized standardized, though slightly different, protocols to transform PBMCs to B-cell lines. Despite differences in relative geographic areas, climatic extremes, and sample transportation challenges, the overall transformation success rate across all the four networks was high (98.4%) with no significant difference in the success rate of the freshly transformed PBMCs versus frozen, re-suspended PBMCs (Table 1). The lower transformation rate observed in the United Kingdom (93.9%) arose from contamination of 21 samples in the final batch. This was due to use of a contaminated batch of PBS buffer purchased as sterile from Inverclyde Biologicals, which later turned out to be nonsterile due to a breach in the sterilization procedure at the factory. This buffer was used to dilute the working stock of cyclosporine A that was added to the cells in the first few days of culture, thereby introducing bacteria to the cultures. It took several weeks to identify the source of the infection by which time a large number of cultures had become contaminated. Prior to the inclusion of these failures in the study database, the transformation rate in the United Kingdom was comparable to the other network repositories (*i.e.,* 97.2%).

**Table 1:** PBMC transformation rates, by network and overall, T1DGC, July 4, 2009

Network	Total samples	Samples transformed	Failed	In process	Success (%)
Asia-Pacific	2208	2050	42	116	98.0
European	4848	4785	61	2	98.7
North American	6422	6191	72	159	98.9
United Kingdom	676	635	41	0	93.9
Overall	14,154	13,661	216	277	98.4

**Table 2:** Summary of sample transit time and cell line transformation outcome, by network and overall, T1DGC, July 4, 2009

		Asia-Pacific	European	North American	United Kingdom	Overall
		*N* (%)	*N* (%)	*N* (%)	*N* (%)	*N* (%)
Transit time (h)	Transformation					
<24	Success	829 (96.9)	2170 (98.8)	3107 (98.8)	184 (98.4)	6290 (98.5)
	Failure	27 (3.2)	26 (1.2)	38 (1.2)	3 (1.6)	94 (1.5)
	Total (%*N*) received	856 (62.6)	2196 (44.9)	3145 (54.4)	187 (26.5)	6384 (50.1)
≥24 and <48	Success	358 (97.6)	2101 (98.4)	1772 (98.9)	260 (91.9)	4491 (98.1)
	Failure	9 (2.5)	35 (1.6)	19 (1.1)	23 (8.1)	86 (1.9)
	Total (%*N*) received	367 (26.9)	2136 (43.7)	1791 (31.0)	283 (40.1)	4577 (35.9)
≥48 and <72	Success	52 (100.0)	205 (99.5)	318 (99.4)	51 (87.9)	626 (98.4)
	Failure	–	1 (0.5)	2 (0.6)	7 (12.1)	10 (1.6)
	Total (%*N*) received	52 (3.8)	206 (4.2)	320 (5.5)	58 (8.2)	636 (5.0)
≥72 and <96	Success	9 (100.0)	61 (100.0)	109 (100.0)	100 (98.0)	279 (99.3)
	Failure	–	–	–	2 (2.0)	2 (0.7)
	Total (%*N*) received	9 (0.7)	61 (1.3)	109 (1.9)	102 (14.5)	281 (2.2)
≥96	Success	78 (94.0)	290 (98.6)	408 (98.3)	69 (92.0)	845 (97.5)
	Failure	5 (6.0)	4 (1.4)	7 (1.7)	6 (8.0)	22 (2.5)
	Total (%*N*) received	83 (6.1)	294 (6.0)	415 (7.2)	75 (10.6)	867 (6.8)
Overall total received		1367 (100.0)	4893 (100.0)	5780 (100.0)	705 (100.0)	12,745 (100.0)

Parameters that might have affected the quality of the blood sample and overall transformation success rate were recorded and tracked for each sample shipment, from recruitment center to DNA repository. These included: transportation time from blood sample collection to receipt at the laboratories; storage conditions of blood samples during transit; quality of blood samples on arrival at the laboratory (*e.g.,* whether blood samples were hemolyzed or clotted or blood tubes were cracked or broken en route); and low volume samples. The T1DGC protocol dictated that the cell line and cell pack samples be shipped on the day of collection for receipt at the DNA Repository within 24 h (optimally). All clinics used courier companies for shipping samples, with the exception of those within the United Kingdom Network where the postal service was used. Deviations from the protocol occurred when clinics did not ship on the day of collection, and/or delays in shipping were encountered by courier companies.

Of the 12,745 samples shipped from clinical sites to the T1DGC DNA Repositories, 6384 (50.1%) were received within 24 h from the time of blood collection (Table 3). Of these, 98.5% were successfully transformed and 1.5% failed. An additional 4577 samples (35.9%) were received by the repositories between ≥24 and <48 h, with 98.1% of these samples successfully transformed and 1.9% failing. The transformation rate for samples in transit between ≥48 and <72 h (*n* = 636; 5.0%) was 98.4%, and it was 99.3% for samples in transit between ≥72 and <96 (*n* = 281; 2.2%). Even for samples that arrived at the repositories more than 4 days after collection (*n* = 867; 6.8%), the transformation rate was 97.5%. There was no correlation between sample transit time and successful transformation, either overall (*r* = 0.02, *p* = 0.07) or within the network.

**Table 3:** Summary of DNA extraction yield from EDTA cell packs, by network and overall, T1DGC, July 4, 2009

Network	Total samples (*N*)	DNA extraction method	Average yield per sample (µg)^a^
Asia-Pacific	2131	Salting out	102.9
European	4836	Salting out	143.0
North American	6393	Salting out	164.9
United Kingdom	662	Chloroform	91.7
Overall	14,022		144.5

a Defined by OD at 260/280 nm.

### Quantity of DNA extracted from whole blood cell packs

Isolation of DNA from the residual cell pack of a 5 mL of whole blood sample (collected in an EDTA vacutainer) was performed using a modified salting out procedure or chloroform extraction, respectively [6,7]. As of July 4, 2009, DNA was extracted from 14,022 EDTA cell pack samples, with an average yield of 144 µg (range: 92–165 µg) (Table 3). Differences in yields across the laboratories reflected differences in the volumes of samples received and protocols used in the laboratories.

**Table 4:** Summary of DNA extraction yield from LCL, by network and overall, T1DGC, July 4, 2009

Network	Total samples (*N*)	Culture vessel surface area (cm^2^)	Average yield per sample (µg)^a^
Asia-Pacific	2077	75	709.4
European	4793	75	390.4
North American	6108	25	237.7
United Kingdom	636	75	266.5
Overall	13,614		364.8

a Defined by OD at 260/280 nm.

### Quantity of DNA extracted from lymphoblastoid cell lines

Isolation of DNA from lymphoblastoid cell lines (LCL) in the four network laboratories using two basic extraction methods [6,7] consistently yielded robust quantities of DNA of extremely high quality (Table 4). As of July 4, 2009, 13,614 LCL samples had been extracted in the four laboratories, with an average yield of 365 µg (range: 238–709 µg) DNA per B-LCL.

**Table 5:** Summary of sample outcomes for classical HLA genotyping, by network and overall, T1DGC, July 4, 2009

	Asia-Pacific *N* (%)	European *N* (%)	North American (Class I) *N* (%)	North American (Class II) *N* (%)	United Kingdom *N* (%)	Total *N* (%)
*N* participants (families)	2029 (579)	4854 (1287)	5822 (1385)	5822 (1385)	668 (169)	13,373 (3420)
MIEs^a^	26 (4.5)	23 (1.8)	32 (2.3)	33 (2.4)	3 (1.8)	92 (2.7)
Failures	3 (0.1)	2 (0.0)	36 (0.6)	36 (0.6)	9 (1.3)	50 (0.4)
Contaminated samples	7 (0.3)	5 (0.1)	13 (0.2)	13 (0.2)	23 (3.4)	48 (0.4)
Replacements	24 (1.2)	22 (0.5)	83 (1.4)	83 (1.4)	42 (6.3)	171 (1.3)

a *N* (%) MIEs is based on number of families.

### Quality of DNA extracted from whole blood cell packs and LCL

**Figure 1:**
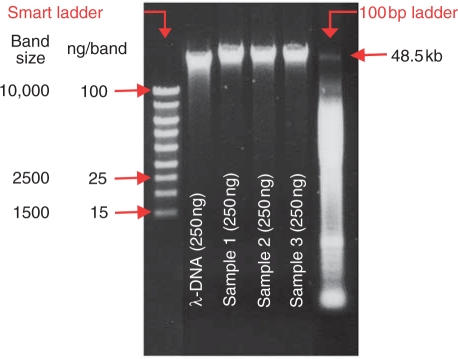
DNA sample quality control. To confirm quality and quantity of the isolated DNA, agarose gel electrophoresis of each DNA sample was performed. By adding the smart ladder, the measured concentration of 250 ng/µL, which was obtained performing OD measurement, could be visually confirmed.

The quality of DNA was assessed by agarose gel electrophoresis. The analysis of each DNA sample extraction by agarose gel electrophoresis, including a molecular weight standard (Invitrogen), showed clear, bright banding at 48.5 kb in >99% of the samples. This is indicative of high molecular weight DNA and absence of DNA degradation. Inclusion of lambda DNA markers (Roche Molecular Systems) in each gel provided additional quantity controls for the test DNA samples and demonstrated comparable sample-by-sample results to the DNA quantified by OD measurement (Figure 1).

### HLA genotyping

In general, the extracted DNA from whole blood cell packs (primary sample source) or PBMCs was of high quality and was successfully utilized in the HLA genotyping method utilized in T1DGC [8]. Overall, only 50 (0.4%) of the 13,373 samples genotyped were noted as failures and an additional 48 (0.4%) as contaminated (Table 6). Contamination could have occurred at either the DNA repository or at the HLA genotyping laboratory during sample aliquoting. Mendelian inheritance errors (MIEs) were found in 92 (2.7%) of the genotyped families. To date, all but four of the MIEs were resolved as follows: 32 (34.8%) were deemed as genotyping errors at the HLA laboratories; 27 (29.3%) were sample mix-ups at the DNA repositories; and 29 (31.5%) were classified as probable nonbiological relationships (*e.g.,* half-siblings, paternity issues). A total of 171 samples (1.3%) were requested as replacement samples by the HLA genotyping laboratories.

**Table 6:** Summary of sample outcomes for genotyping projects, by network and overall, T1DGC, July 4, 2009

	Asia-Pacific *N* (%)	European *N* (%)	North American *N* (%)	United Kingdom *N* (%)	Total *N* (%)
6K Genome scan (CIDR)					
*N*	1338	4741	4813	631	11,523
MIEs	7 (0.5)	32 (0.7)	27 (0.6)	2 (0.3)	68 (0.6)
Failures	8 (0.6)	28 (0.6)	65 (1.4)	10 (1.6)	111 (1.0)
Gender discrepancies	2 (0.1)	7 (0.1)	2 (0.0)	0 (0.0)	11 (0.1)
Sample switches	16 (1.2)	12 (0.3)	40 (0.8)	0 (0.0)	68 (0.6)
Replacements	9 (0.7)	66 (1.4)	109 (2.3)	13 (2.1)	197 (1.7)
MHC fine mapping (The Wellcome Trust Sanger Institute)					
*N*	782	1953	1373	484	4592
MIEs	4 (0.5)	13 (0.7)	17 (1.2)	6 (1.2)	40 (0.9)
Failures – OPA1	5 (0.6)	15 (0.8)	14 (1.0)	4 (0.8)	38 (0.8)
Failures – OPA2	6 (0.8)	3 (0.2)	9 (0.7)	14 (2.9)	32 (0.7)
Failures – microsatellites	13 (1.6)	38 (1.9)	20 (1.5)	12 (2.5)	83 (1.8)
Gender discrepancies	0 (0.0)	0 (0.0)	0 (0.0)	2 (0.4)	2 (0.0)
Sample switches	4 (0.5)	6 (0.3)	6 (0.4)	2 (0.4)	18 (0.4)
Replacements	18 (2.3)	29 (1.5)	39 (2.8)	31 (6.4)	117 (2.5)
Rapid response (The Broad Institute)					
*N*	782	1953	1376	484	4595
MIEs	5 (0.6)	13 (0.7)	9 (0.7)	8 (1.7)	35 (0.8)
Failures – llumina	19 (2.4)	12 (0.6)	53 (3.9)	42 (8.7)	126 (2.7)
Failures – Sequenom	26 (3.3)	55 (2.8)	75 (5.5)	25 (5.2)	181 (3.9)
Gender discrepancies	0 (0.0)	0 (0.0)	1 (0.1)	2 (0.4)	3 (0.1)
Sample switches	2 (0.3)	6 (0.3)	8 (0.6)	0 (0.0)	16 (0.3)

### Additional genotyping projects

In addition, the T1DGC utilized several laboratories, including the Center for Inherited Disease Research (CIDR, Johns Hopkins University, Baltimore, MD, USA), the Wellcome Trust Sanger Institute (Cambridge, UK), and the Broad Institute (Massachusetts Institute of Technology/Harvard University, Cambridge, MA, USA) to complete the genotyping of thousands of samples from T1DGC as well as other existing collections from a variety of populations. A general assessment of the overall quality of the T1DGC DNA samples submitted as well as potential sample labeling or aliquoting errors can be obtained by examining sample failures, MIEs, sample switches, and gender discrepancies for each of the genotyping projects (Table 6). Sample labeling errors could have occurred at the clinic collection site during sample collection or at the DNA repository during sample preparation or aliquoting. Aliquoting errors also could have occurred at either the DNA repository or the genotyping facility (if samples were re-aliquoted prior to or during the production phase of the project).

Across all the four T1DGC repositories, DNA was extracted from both LCLs and whole blood cell packs. The isolated DNA was then sent to a variety of genotyping laboratories for analysis by various methods, using a number of different platforms. The genotyping results obtained from the various laboratories, using the isolated DNA, were all excellent, regardless of the platform used [9–11].

#### Genome-wide 6K single nucleotide polymorphism analysis (CIDR)

The T1DGC repositories submitted 11,523 samples with 10 µg of genomic DNA (100 µL at 100 ng/mL) to CIDR for 6K genome-wide single nucleotide polymorphism (SNP) analysis. Samples were aliquoted into 96-well plates supplied by CIDR, with two quality control samples and two empty wells (for control samples) per plate, for genotyping using the Illumina Human Linkage-12 Genotyping Beadchip consisting of 6090 SNPs on the Illumina platform. Of the production samples submitted, only 111 (1.0%) were noted as failures. Sixty-eight samples (0.6%) resulted in MIEs; 11 (0.1%) indicated gender discrepancies (as compared with the phenotypic data from data collection forms); and 68 (0.6%) indicated sample switches. A total of 197 replacement samples (1.7%) were requested.

#### Major histocompatibility complex fine mapping project (The Wellcome Trust Sanger Institute)

The T1DGC repositories submitted 4592 samples with 10 µg of genomic DNA (100 µL at 100 ng/mL) to Sanger for genotyping analysis on two 1536 SNP oligonucleotide pool assays (OPAs) and 63 microsatellites in the major histocompatibility complex (MHC) region. Samples were aliquoted into 96-well plates, with two quality control samples and one empty well (for control samples) per plate, for genotyping on the Illumina platform; microsatellites were genotyped at deCODE (Iceland). Of the production samples submitted, only 38 (0.8%) were noted as failures on OPA1 and 32 (0.7%) on OPA2. Sample failures were slightly higher for microsatellites (1.8%). Forty samples (0.9%) resulted in MIEs; two (0.0%) indicated gender discrepancies; and 18 (0.4%) indicated sample switches. A total of 117 replacement samples (2.5%) were requested.

#### Rapid response (The Broad Institute)

The T1DGC repositories submitted 4595 samples with 5 µg of genomic DNA (50 µL at 100 ng/mL) to the Broad Institute for 384 SNP analysis on two platforms (Illumina and Sequenom). Samples were aliquoted into 96-well plates, with two quality control samples and one empty well (for control samples) per plate. Of the production samples submitted, there were 126 (2.7%) failures on the Illumina platform and 181 (3.9%) on the Sequenom platform. Thirty-five samples (0.8%) resulted in MIEs; three (0.1%) indicated gender discrepancies; and 16 (0.3%) indicated sample switches.

## Discussion

The T1DGC was established to unite several groups of investigators worldwide who shared the common goal of identifying genes relating to the etiologies of type 1 diabetes mellitus. The recruitment protocols of the T1DGC allowed many more probands, relatives, and families to be recruited than was previously possible in country- or region-wide efforts. Establishing regional DNA repositories was a logical response to worldwide recruitment. Implementation of the repositories allowed these central processes to be located near the clinical centers, thus minimizing time in shipment. Other advantages subsumed dissemination of technical expertise, including benefits of scientific cooperation among several regions, distribution of workload for genotyping projects among several laboratories, rapid and efficient distribution of samples to investigators, and utilization of validated quality control procedures throughout the Consortium.

To yield transformed cells, whole blood samples were collected and sent the same day by overnight courier (or equivalent) to the laboratory for the Asia-Pacific, European, and North American Repositories. The Asia-Pacific and European Repositories processed all samples to the point of initiating the transformation cultures on the day they were received, which often necessitated a long working day to complete the processing. The North American and United Kingdom Repositories processed the samples to the point of PBMC isolation only, with the PBMCs being frozen for later transformation. This allowed the initial processing of the blood specimens to be divided into two phases, reducing the overall time required for processing on the day of receipt as well as permitting the ‘batch’ transformation of frozen–thawed PBMCs. This latter strategy had the advantage of batch-processing time savings and the synchronization of transforming cultures.

Either modality worked satisfactorily in the T1DGC. Thus, the long distances for shipping in Asia-Pacific did not reduce the yield of the transformed cells. Among the four laboratories, the different processes outlined above generally produced quite similar results (*i.e.,* the success in transformation was very high and nearly identical among the repositories). The explanations for transformation failures (*i.e.,* collection vagaries, transportation issues, and intra-laboratory problems) were similar among the regions. Although we expected Asia-Pacific to encounter the greatest challenges due to the long distances required for shipping, their transformation rates were very good and similar to those achieved by others. These results support the resilience of cell line samples as well as the ability of the repositories to achieve transformation when delivery to the laboratory was delayed.

The variation in cell line DNA yields among the laboratories may have been in part related to the different DNA extraction protocols used. However, it is also possible that the primary source of variation was due to the different culture vessels with different volumes for culture employed for growing the cell lines (*i.e.,* flasks with 25 cm^2^ vs 75 cm^2^ surface area), and thus the number of PBMCs per flask subjected to DNA extraction.

The provision of large amounts of DNA is the central goal of many different studies. We established a robust and workable model that other large multi-center, multi-regional epidemiologic studies, or clinical trials can follow. We measured the amounts of DNA, aliquoted DNA for additional testing successfully completed in many facilities, and checked for errors at many steps in the process. In its final accomplishments, the T1DGC established a centralized cell bank that will continue to provide a reference for diabetes research. The availability of well-characterized, viable lymphoblastoid human cells, which can also be used for functional studies along with pertinent data on the specific patient group, is a renewable resource that will permit state-of-the-art biomedical research.

T1DGC quality control programs guided each of the laboratories and genotyping facilities and facilitated efficient completion of the protocols and procedures. Overall, the results demonstrate success at all levels of endeavor: from collection to shipping to processing and finally to analyses and reporting to the Coordinating Center. Furthermore, the quality control checks for quality and consistency of materials and assays allowed the T1DGC to document the success of the enterprise: provision of high-quality DNA for genotyping on many different platforms for contrasting and intersecting goals to elucidate the genetics of type 1 diabetes. Finally, the careful documentation within the T1DGC showed what parameters were important for the success of the Consortium.
